# Lateral Lymph Node Metastases in T1a Papillary Thyroid Carcinoma: Stratification by Tumor Location and Size

**DOI:** 10.3389/fendo.2021.716082

**Published:** 2021-07-15

**Authors:** Xiaojun Zhang, Wenkuan Chen, Qigen Fang, Jie Fan, Lu Feng, Lanwei Guo, Shanting Liu, Hong Ge, Wei Du

**Affiliations:** ^1^ Department of Head Neck and Thyroid Surgery, The Affiliated Cancer Hospital of Zhengzhou University, Henan Cancer Hospital, Zhengzhou, Henan, China; ^2^ Department of Head and Neck Surgery, State Key Laboratory of Oncology in South China, Collaborative Innovation Center for Cancer Medicine, Sun Yat-sen University Cancer Center, Guangzhou, China; ^3^ Office for Cancer Control and Research, Henan Cancer Hospital, The Affiliated Cancer Hospital of Zhengzhou University, Zhengzhou, China; ^4^ Department of Radiation Oncology, The Affiliated Cancer Hospital of Zhengzhou University, Henan Cancer Hospital, Zhengzhou, Henan, China

**Keywords:** papillary thyroid carcinoma, lymph node metastases, upper lobe, tumor location, tumor size

## Abstract

**Objective:**

To analyze the incidence and risk factors for lateral lymph node metastases (LNMs) in T1a papillary thyroid carcinoma (PTC) with a focus on tumor location and size.

**Materials and Methods:**

The incidence of lateral LNM in 345 cases of T1a PTC was retrospectively analyzed. Univariate and multivariate analyses were performed to assess the relationships between lateral LNM and clinicopathological characteristics.

**Results:**

The incidence of skip metastasis to lateral LNM in T1a PTC located in the upper lobe was 12.1% (8/66). Logistic regression analysis indicated tumor size >5 mm (OR = 5.04, 95% CI = 1.79 to 14.18, P = 0.002), upper lobe location (OR = 7.68, 95% CI = 3.05–19.34, P < 0.001) and the number of central neck LNM (<2: OR = 24.79, 95% CI = 8.23–74.60, P < 0.001; ≥2: OR = 4.99, 95% CI = 1.95–12.73, P < 0.001) were independently associated with lateral LNM. Comparing the lateral and central LNM stratification based on tumor location revealed that both the incidences of lateral (33.3%) and central (30.3%) LNM of T1a PTC located in the upper lobe were higher than those of T1a PTC located in the middle and lower lobes. Of T1a PTC located in the upper lobe, the incidence of lateral LNM was 33.3% (22/66), which was higher than that [30.3% (20/66)] of central LNM. This finding is reversed in all T1a PTC cases and T1a PTC cases with tumor located in the middle and lower lobes.

**Conclusion:**

A particularly high likelihood of lateral LNM was observed in T1a PTC patients with tumor located in the upper lobe of the thyroid gland, especially the tumor >5 mm in size, which could be considered a risk factor for lateral LNM in the clinical management of T1a PTC.

## Introduction

The incidence of papillary thyroid carcinoma (PTC), especially occurrences smaller than 10 mm, continues to increase, arousing worldwide concern (1–5). A papillary thyroid carcinoma measuring ≤10 mm in its greatest dimension is defined as papillary thyroid microcarcinoma (PTMC) according to the World Health Organization classification system for thyroid tumors (6). The risk factors for the aggressiveness of PTMC are extrathyroidal extension (ETE) and clinically positive cervical lymph nodes (3). For PTMC without risk factors for aggressiveness, active surveillance can be considered an alternative option due to its indolent nature in biology (3, 7–10). For PTMC with risk factors for aggressiveness, surgery should be implemented in a limited time frame.

However, unlike ETE, it is typically not easy to assess the lymph node metastases (LNM) of PTMC on US or CT in clinical practice, especially in some hospitals without high-resolution US and thin-layer CT in developing countries. At present, there is a great difference in the study of the incidence of lateral LNM of PTMC. Zhao (11) found that lateral LNM was observed in 163 (75.8%) of 215 PTMC patients. However, Kim (12) found that approximately 4.4% of patients with PTMC presented with lateral LNM at the time of diagnosis. The effect of ETE on lateral LNM and prognosis has been well established (3, 13–17). Nevertheless, there are few studies on lateral LNM in PTMC without ETE. In this study, we focused on analyzing the lateral LNM of PTMC without ETE, that is, T1a PTC.

## Patients and Methods

### Ethics Statement

The Hospital Institutional Research Committee approved our study, and all participants provided written informed consent. All methods were performed in accordance with the relevant guidelines and regulations. All procedures in studies involving human participants were performed in accordance with the ethical standards of the institutional and/or national research committee and with the 1964 Declaration of Helsinki and its later amendments or comparable ethical standards.

### Patient Selection

From January 2017 to May 2017, the medical records of patients with surgically treated primary PTC were retrospectively reviewed. Enrolled patients met the following criteria: clinicopathological characteristics, including the location, size, multifocality, extrathyroidal extension of the tumor; lateral and central neck LNM; and BRAF^V600E^ mutation status. Data were recorded in detail. PTMC was confirmed by pathologic analysis; that is, PTC measuring ≤10 mm in its greatest dimension (the largest disease if it is a multifocal PTC). Then, 367 PTMC patients were selected.

According to the American Joint Committee on Cancer (AJCC) TNM Staging For Thyroid-Medullary Carcinoma (8th ed., 2017), PTC with only minimal extrathyroidal extension invading strap muscles did not belong to T3b stage, so 16 cases with gross extrathyroidal extension invading strap muscles (T3b) and 6 cases with gross extrathyroidal extension into the nearby tissues of the neck, including the larynx, trachea, esophagus, or recurrent laryngeal nerve (T4a), were excluded. Therefore, a total of 345 T1a PTC patients were enrolled for analysis.

### Treatment Principles

Based on 2016 NCCN guidelines for thyroid cancer (3), of 345 T1a PTC patients in this study, 81 patients received total thyroidectomy and 264 patients received lobectomy. Ipsilateral central neck lymph node dissection (LND) was routinely performed on PTC patients treated for the first time. In the lateral compartment, a formal modified radical LND including levels II, III, IV, and Vb was performed for pathologically positive lateral neck LNM (**Table 1**).

**Table 1:** Demographics and clinicopathologic characteristics of patients.

Characteristic		Value	Percentage (%)
Age	<55 year	282	81.7
	≥55 years	63	22.3
Gender	Male	81	23.5
	Female	264	76.5
Multifocality	Yes	103	29.9
	No	242	70.1
HT	Yes	118	34.2
	No	227	65.8
Tumor size	≤5 mm	147	42.6
	>5 mm, ≤10 mm	198	57.4
Tumor location	Upper lobe	66	19.1
	Middle lobe	196	56.8
	Lower lobe	83	24.1
Primary treatment	Total thyroidectomy	81	23.5
	Lobectomy	264	76.5
	Central LND	345	100.0
	Lateral LND	44	12.8
Central LNM (n*)	0	264	76.5
	≤2	54	15.7
	>2	27	7.8
Lateral LNM	Yes	44	12.8
	No	301	87.2
BRAF^V600E^	Mutation	283	82.0
	Wild	62	18.0

*number of lymph node metastases.

**Figure 1:**
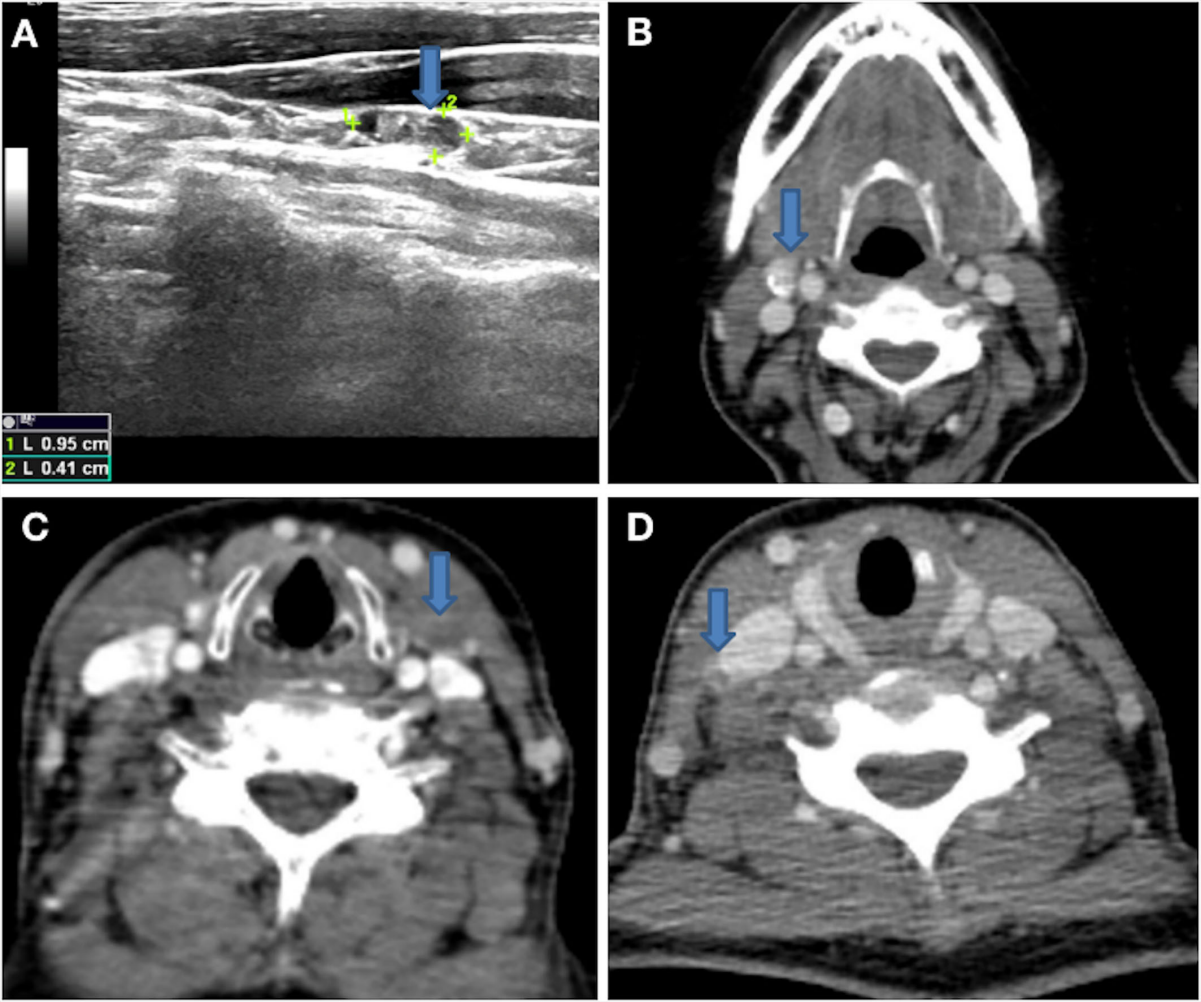
**(A)** Lateral neck lymph node with microcalcification on high-resolution US. **(B)** Lateral neck lymph node with microcalcification on 1-mm thin layer contrast-enhanced CT. **(C, D)** Lateral neck lymph nodes with low-density or liquid components on 1-mm thin layer contrast-enhanced CT.

Pathologically positive lateral neck LNM was proven by preoperative biopsy or intraoperative frozen pathology with clinically positive lateral neck LNM. Clinical positive lateral neck LNM was suggested if the following features were noted on high-resolution ultrasound (US): absence of an echogenic hilum, round shape, microcalcification (**Figure 1A**), peripheral blood flow on color Doppler images, or cystic changes (18, 19). Clinical positive lateral LNM was suggested if the following features were noted on computed tomography (CT): area with clear evidence of non-fat, microcalcification (**Figure 1B**), low-density, or liquid components (**Figures 1C, D**); largest diameter >15 mm at level II and >10 mm at other levels; and the ratio of the longest to smallest diameter ≤ 2.

### Statistical Analysis

We used descriptive statistics to summarize patient demographics and the general characteristics of thyroid tumors. Data are presented as frequencies (percentages) for categorical variables with means (standard deviations) or (range) as appropriate for continuous variables.

The following factors were examined as possible factors associated with lateral LNM in T1a PTC: sex, age (<55 *vs* ≥55 years old), Hashimoto’s thyroiditis, location, size, multifocality of the tumor, number of central LNM and BRAF^V600E^ mutation status. Tumor size was treated as a continuous measurement (in mm) and as a binary categorical variable with two categories (≤5 *vs >*5 mm). Based on US and CT images, the tumor location was categorized as upper lobe, middle lobe, or lower lobe. The thyroid was subjectively divided into thirds to differentiate the upper, middle, and lower lobes. Thyroid nodules occupying both the middle and upper thyroid lobes were included in the upper thyroid nodule category. Thyroid nodules occupying both the middle and lower thyroid lobes were included in the lower thyroid nodule category, as described in an early study (20). In addition, isthmus tumors were included in the middle lobe in this study, and the largest lesion was assessed if the tumor was multifocal. The number of central LNMs was treated as a continuous measurement and as a binary categorical variable with three categories (0 *vs ≤*2 *vs >*2).

Univariate analysis was performed using Pearson’s chi-square test; statistically significant results obtained from univariate analysis were submitted to multivariate logistic regression. The association of lateral LNM with each factor was expressed as odds ratios (ORs) and 95% confidence intervals (CIs). All statistical analyses were performed with SPSS 20.0 and P <0.05 was considered to be significant.

## Results

### Clinical and Pathologic Characteristics of Patients With T1a PTC

Ultimately, 345 T1a PTC patients (81 males and 264 females) were enrolled with a mean age of 47.0 (range: 20–74) years. Eighty-one (23.5%) patients underwent total thyroidectomy for bilateral tumors or lateral LNM, 81 (23.5%) patients had LNM, 44 (12.8%) patients had lateral neck LNM, 52 (15.1%) patients had bilateral thyroid lobe tumors, and 283 (82.0%) patients had BRAF^V600E^ mutations (**Table 1**).

Of all T1a PTCs, the incidence of skip metastases to lateral LNM with central lymph node negativity was 4.3% (15/345), whereas that of T1a PTC with tumors located in the upper lobe with skip metastases was 12.1% (8/66).

### Univariate Analysis of Factors Related to Lateral LNM in 345 Cases of T1a PTC

To explore the factors of lateral LNM of T1a PTC, the correlation between lateral LNM of T1a PTC and possible related clinicopathologic factors was further analyzed. The univariate analysis (**Table 2**) showed a statistically significant association between lateral LNM of T1a PTC and tumor size (≤5 mm *vs >*5 mm, P < 0.001), multifocality (P = 0.015), tumor location (upper, middle, and lower parts of thyroid lobe, P < 0.001) and the number of central LNM (>2, P < 0.001). No difference was observed between lateral LNM of T1a PTC and age (P = 0.156), sex (P = 0.162), Hashimoto’s thyroiditis (P = 0.987) or BRAF^V600E^ (P = 0.085).

**Table 2:** Univariate analyses of factors related to lateral LNM in 345 cases of T1a PTC.

Characteristic		Cases	Lateral LNM		χ^2^	*P*
			Yes	No		
Age	<55 years	282	35	247	0.16	0.687
	≥55 years	63	9	54		
Gender	Male	81	14	67	1.95	0.162
	Female	264	30	234		
Multifocality	Yes	103	20	83	5.86	0.015
	No	242	24	218		
HT	Yes	118	15	103	<0.01	0.987
	No	227	29	198		
Tumor size	≤5 mm	147	6	141	17.31	<0.001
	>5 mm, ≤10 mm	198	38	160		
Tumor	Upper lobe	66	22	44	31.10	<0.001
location	Middle lobe	196	15	181		
	Lower lobe	83	7	76		
Central	0	264	15	249	85.21	<0.001
LNM (n*)	≤ 2	54	11	43		
	> 2	27	18	9		
BRAF^V600E^	Mutation	283	32	251	2.96	0.085
	Wild	62	12	50		

*number of lymph node metastasis.

HT, Hashimoto’s thyroiditis.

### Multivariate Analysis of Factors Related to Lateral LNM in 345 Cases of T1a PTC

The results of the logistic regression analysis are shown in **Table 3** and revealed three significant predictors for lateral neck LNM in T1a PTC: tumor size >5 mm (OR = 5.04, 95% CI = 1.79 to 14.18, P = 0.002), upper lobe location (OR = 7.68, 95% CI = 3.05–19.34, P < 0.001) and the number of central neck LNM (<2: OR = 24.79, 95% CI = 8.23–74.60, P < 0.001; ≥2: OR = 4.99, 95% CI =1.95–12.73, P < 0.001). However, the risk factor multifocality was not a significant predictor for lateral neck LNM in T1a PTC (OR = 2.00, 95% CI = 0.90–4.46, P < 0.001).

**Table 3:** Multivariate analysis of factors related to lateral LNM in 345 patients with T1a PTC.

Variable	*β*	Std	Wald χ^2^	OR (95% CI)	*P*
**Tumor size**					
≤5mm				1.00	
>5 mm ≤10 mm	1.62	0.53	9.40	5.04 (1.79–14.18)	0.002
**Multifocality**	0.69	0.41	2.87	2.00 (0.90–4.46)	0.090
**Tumor location**					
Upper lobe	2.04	0.47	18.72	7.68 (3.05–19.34)	<0.001
Middle lobe			21.78	1.00	
Lower lobe	−0.35	0.556	0.004	0.97 (0.33–2.87)	0.950
**Central LNM**					
0			34.71	1.00	
≤2	1.61	0.48	11.29	4.99 (1.95–12.73)	0.001
>2	3.21	0.562	32.586	24.79 (8.23–74.60)	<0.001
**Constant**	−5.01	0.736	46.38		<0.001

### Incidence of Lateral LNM in T1a PTC Patients Stratified by Tumor Location And Size

**Figure 2:**
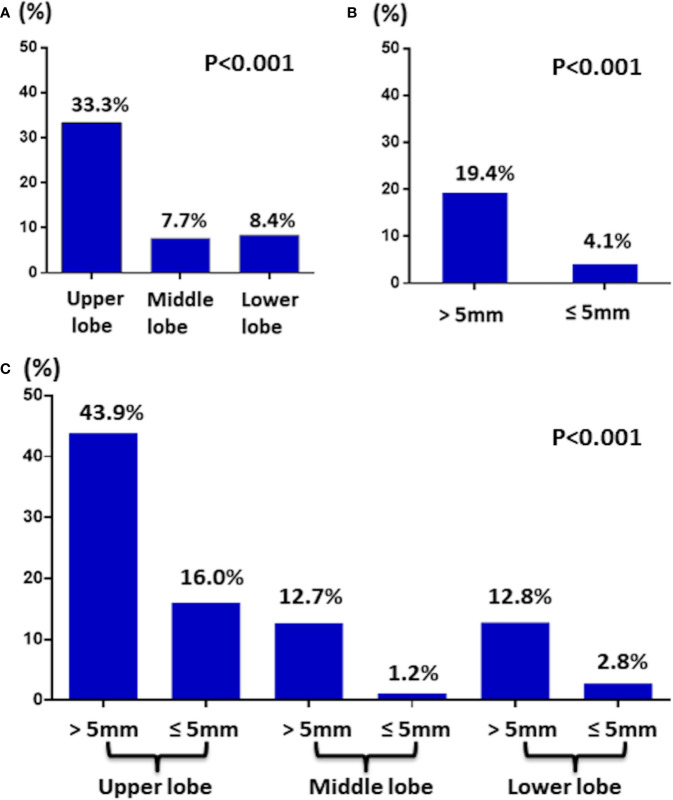
**(A)** The incidence of lateral LNM in T1a PTC by tumor location. **(B)** The incidence of lateral LNM in T1a PTC by tumor size. **(C)** The incidence of lateral LNM in T1a PTC by tumor location and size.

Of 345 T1a PTC patients, 44 (12.8%) patients had lateral neck LNM. Stratification by location revealed that of 66 T1a PTC patients had tumors located in the upper part, 22 (33.3%) patients had lateral neck LNM, which was greater than 7.7% (15/196) in the middle part and 8.4% (7/83) in the lower part (**Figure 2A**).

Then, stratification by size revealed that of 198 T1a PTC patients with tumors >5 mm, 38 (19.4%) patients had lateral LNM, which was greater than the 4.1% (6/147) noted for patients with tumors ≤5 mm (**Figure 2B**). Interestingly, of six T1a PTC patients with tumors ≤5 mm and lateral LNM, four tumors of the patients were located in the upper part.

Finally, stratification by location together with size revealed that of 41 T1a PTC patients with tumor >5 mm and located in the upper part, 18 (43.9%) patients had lateral LNM (**Figure 2C**).

### Comparison of Lateral LNM and Central LNM Stratification by Tumor Location

As shown in **Table 4**, 23.5% (81/345) of all T1a PTCs patients had central LNM, and 12.8% (44/345) of patients had lateral LNM. We compared lateral and central LNM stratified by tumor location and found that both the incidence of lateral (33.3%) and central (30.3%) LNM of T1a PTC with tumor located in upper lobe were higher than that of T1a PTC with tumor located in middle and lower lobes; of T1a PTC with tumor located on upper lobe, the incidence of lateral LNM was 33.3% (22/66), which is slightly higher than that noted for central LNM [30.3% (20/66)]. This finding is reversed in all T1a PTC and T1a PTC with tumors located in the middle and lower lobes.

**Table 4:** The incidence of central and lateral LNM in T1a PTC by tumor location.

Incidence (%)	Central LNM	Lateral LNM
Tumor location		
Upper lobe	**30.3% (20/66)**	**33.3% (22/66)**
Middle lobe	19.4% (38/196)	7.7% (15/196)
Lower lobe	26.5% (23/83)	8.4% (7/83)
Overall	23.5% (81/345)	12.8% (44/345)

In bold: emphasizing the values.

### Comparison of Lateral LNM and Central LNM Stratification by Age

As shown in **Table 5**, we compared the incidence of lateral and central LNMs of T1a PTC stratified by age and found that the incidences of central LNM of T1a PTC with age <55 and ≥55 years were 22.0% (62/282) and 30.2% (19/63) respectively (P = 0.166); the incidences of lateral LNM of T1a PTC with age <55 and ≥55 years were 12.4% (35/282) and 14.3% (9/63) respectively (P = 0.687). The incidence of central LNM of T1a PTC with age ≥55 year was slightly higher than that of T1a PTC with age <55; however, there was no statistical difference.

**Table 5:** The incidence of central and lateral LNM in T1a PTC by age.

Incidence (%)	Central LNM	Lateral LNM
Age		
<55 years	22.0% (62/282)	12.4% (35/282)
≥55 years	30.2% (19/63)	14.3% (9/63)
Overall	23.5% (81/345)	12.8% (44/345)

## Discussion

The most important finding in the current study was that the rate of lateral LNM of T1a PTC with tumor located in the upper lobe was 33.3%, which is distinctly higher than that of T1a PTC with tumor located in the middle lobe (7.7%) and lower lobes (8.4%). Additionally, the rate of lateral LNM of T1a PTC with tumor >5 mm was 19.4%, which was also higher than that of T1a PTC with tumor ≤5 mm (4.1%). Finally, when considering location together with size, we found that the incidence reached 43.9% in T1a PTC patients with tumor >5 mm and located in the upper lobe.

Cervical LNM in PTC is common and is detected in up to 80% of patients as a result of radical neck dissection and postoperative histological examination (21). Kim (22) found that the overall frequencies of central and lateral LNMs in PTMC were 39.6% and 3.0%, respectively. A meta-analysis of 5,342 PTC cases showed that (23) the frequency of occult lateral neck lymph node metastases was 47.8%. A majority of studies have shown that cervical LNM, especially lateral LNM, carries prognostic significance (21, 24–26). When excluding invasive PTMC (T3–4) in this study, the overall frequencies of central and lateral LNMs in T1a PTC were 23.5% (81/345) and 12.8% (44/345), respectively.

Of T1a PTCs in this study, the incidence of skip metastases was 4.3% (15/345), whereas that of T1a PTC in the upper lobe was 12.1% (8/66). Zhao (27) reviewed 721 PTCs and found that the rate of skip metastases was 7.4%, and PTMC was an independent risk factor for skip metastases.

In this study, we found that lateral LNM of T1a PTC was associated with tumor size (≤5 mm *vs >*5 mm, P < 0.001), multifocality (P = 0.015), tumor location (upper, middle and lower lobes, P < 0.001), and central LNM. Further logistic regression analysis indicated that tumor size >5 mm (OR = 5.04, 95% CI = 1.79 to 14.18, P = 0.002), upper lobe location (OR = 7.68, 95% CI = 3.05–19.34, P < 0.001) and the number of central neck LNM (<2: OR = 24.79, 95% CI = 8.23–74.60, P < 0.001; ≥2: OR = 4.99, 95% CI = 1.95–12.73, P < 0.001) were significant predictors for lateral LNM in T1a PTC.

Similar to our results, Liu (28) reviewed 966 PTCs and found that only central LNM was independent risk factors for lateral LNM, but he also indicated that tumor located in the upper 1/3 of the lobe had the highest lateral LNM incidence (60%). Back (29) found that superior lesions, male sex, age under 45 years, and central lymph node metastasis were significant predictors of high-risk lateral LNM. Zhang (30) found that male sex, upper location of the tumor and tumor diameter ≥0.7 cm were predictive factors of lateral LNM in solitary PTMC without gross ETE. Kim (31) found that male sex, tumor size >0.5 cm, multiplicity, ETE, and central LNM were independent predictors of a high prevalence of lateral LNM in PTMC. In another study, multivariate analysis indicated that the significant risk factors for lateral LNM included ETE, multifocality, and central node metastases but not BRAF mutation (12).

Intriguingly, one of the major findings of the current study is the relatively high prevalence (33.3%) of lateral LNM of T1a PTC with tumors located in the upper lobe. Some studies (29, 30, 32–36) also indicated that the superior lesion or upper location of the tumor was an independent predictor for lateral LNM of PTMC with or without gross ETE. However, it is not entirely clear why the tumor location (upper lobe) is independently associated with lateral LNM in T1a PTC because the effects of ETE and tumor size were already excluded. This finding is probably due to the unique anatomical structure of the upper lobe, which has an abundant blood supply and lymphatic drainage. It is also likely that lymphatic vessels exist between the upper lobe of the thyroid and lateral neck by which tumor cells can metastasize directly to the lateral neck (37). In our observation, the proposed mechanisms may also include persistent physical pressure from adjacent thyroid cartilage. Recently, two small studies proposed integrating nodule location within the thyroid gland into risk stratification systems (20, 37, 38). In the two prospective clinical trials of active surveillance for low-risk PTMC from Japanese centers, a small number of PTMCs under active surveillance showed progression of tumor enlargement or new LNM appearance (39, 40). It is interesting to analyze whether the appearance of newly appeared LNM was associated with the tumor located in the upper lobe.

Our study has some limitations. First, there is inherent bias in all retrospective studies. Second, our sample size was relatively small, which potentially reduced the statistical power of this study. Third, lateral lymph node dissection is performed only when the suspected lateral lymph node is pathologically proven; thus, some micrometastases may be missed. Some merits of this study should also be noted. First, preoperative and lateral lymph nodes were accurately evaluated through high-resolution US and 1-mm thin layer contrast-enhanced CT (**Figure 1**), which was performed by a multidisciplinary team (MDT) composed of a surgeon, sonographer, radiologist, and pathologist specializing in thyroid carcinoma. Second, the characteristics of lateral LNM of T1a PTC were described in detail, especially the tumor located in the upper lobe.

In conclusion, the major findings of the current study are the relatively high incidence (33.3%) of lateral LNM of T1a PTC with tumor located in the upper lobe. Furthermore, when considering tumor size together with location, the incidence reached 43.9% in T1a PTC patients with tumor >5mm and located in the upper lobe.

## Data Availability Statement

The raw data supporting the conclusions of this article will be made available by the authors, without undue reservation.

## Ethics Statement

Written informed consent was obtained from the individual(s), and minor(s)’ legal guardian/next of kin, for the publication of any potentially identifiable images or data included in this article.

## Author Contributions

All the authors made the contribution in study design, manuscript writing, studies selecting, data analysis, study quality evaluating, and manuscript revising. All authors contributed to the article and approved the submitted version.

## Conflict of Interest

The authors declare that the research was conducted in the absence of any commercial or financial relationships that could be construed as a potential conflict of interest.
