# Corrigendum: Lateral Lymph Node Metastases in T1a Papillary Thyroid Carcinoma: Stratification by Tumor Location and Size

**DOI:** 10.3389/fendo.2022.852751

**Published:** 2022-02-01

**Authors:** Xiaojun Zhang, Wenkuan Chen, Qigen Fang, Jie Fan, Lu Feng, Lanwei Guo, Shanting Liu, Hong Ge, Wei Du

**Affiliations:** ^1^ Department of Head Neck and Thyroid Surgery, The Affiliated Cancer Hospital of Zhengzhou University, Henan Cancer Hospital, Zhengzhou, Henan, China; ^2^ Department of Head and Neck Surgery, State Key Laboratory of Oncology in South China, Collaborative Innovation Center for Cancer Medicine, Sun Yat-sen University Cancer Center, Guangzhou, China; ^3^ Office for Cancer Control and Research, Henan Cancer Hospital, The Affiliated Cancer Hospital of Zhengzhou University, Zhengzhou, China; ^4^ Department of Radiation Oncology, The Affiliated Cancer Hospital of Zhengzhou University, Henan Cancer Hospital, Zhengzhou, Henan, China

**Keywords:** papillary thyroid carcinoma, lymph node metastases, upper lobe, tumor location, tumor size

In the originally published article, affiliations 1 and 4 were presented incorrectly.

Affiliation 1 was presented as “Department of Head Neck and Thyroid Surgery, Henan Cancer Hospital, The Affiliated Cancer Hospital of Zhengzhou University, Zhengzhou, Henan, China”; it should be “Department of Head Neck and Thyroid Surgery, The Affiliated Cancer Hospital of Zhengzhou University, Henan Cancer Hospital, Zhengzhou, Henan, China”

Affiliation 4 was presented as “Department of Radiation Oncology, Henan Cancer Hospital, The Affiliated Cancer Hospital of Zhengzhou University, Zhengzhou, Henan, China”; it should be

“Department of Radiation Oncology, The Affiliated Cancer Hospital of Zhengzhou University, Henan Cancer Hospital, Zhengzhou, Henan, China”.

The authors apologize for this error and state that this does not change the scientific conclusions of the article in any way. The original article has been updated.

## Publisher’s Note

All claims expressed in this article are solely those of the authors and do not necessarily represent those of their affiliated organizations, or those of the publisher, the editors and the reviewers. Any product that may be evaluated in this article, or claim that may be made by its manufacturer, is not guaranteed or endorsed by the publisher.

